# Engineering the surface chemistry of silicon nanocrystals for efficient Ag-Si hybrid photocatalysts toward CO_2_ reduction

**DOI:** 10.3389/fchem.2026.1799409

**Published:** 2026-05-12

**Authors:** Huai Chen, Xuebiao Deng, Meiqi Lin, Lei Liang, Tingting Chen, Jiale Si, Yangyang Xiong, Zhenyu Yang, Linwei Yu

**Affiliations:** 1 MOE Laboratory of Bioinorganic and Synthetic Chemistry, Lehn Institute of Functional Materials, School of Chemistry, Sun Yat-sen University, Guangzhou, China; 2 State Key Laboratory of Silicon and Advanced Semiconductor Materials, ZJU-Hangzhou Global Scientific and Technological Innovation Center, School of Materials Science and Engineering, Zhejiang University, Hangzhou, China

**Keywords:** CO_2_ reduction, photocatalysts, silicon nanocrystals (SiNCs), silver nanopartcles, surface chemistry

## Abstract

Plasmonic nanoparticles (NPs) enable exceptional light harvesting via localized surface plasmon resonances (LSPR), yet their photocatalytic utility is limited by ultrafast charge recombination and surface contamination from synthesis. Here, we overcome these challenges by applying the surface chemistry of silicon nanocrystals (SiNCs) to synthesize hybrid nanostructures of uncoated silver nanoparticles (AgNPs). The alkyl-passivated SiNC surface acts as a reductive template, guiding the *in-situ* formation of clean, interfacially coupled AgNPs while protecting the SiNC core from oxidation. This tailored architecture leverages a synegiestic mechanism to enhance AgNP plasmon resonance, drive efficient hot-electron transfer and improve charge carrier separation. The resulting Ag@SiNC hybrid achieves improved CO_2_ reduction performance, with a CO yield of 1,552 μmol/g and near 100% selectivity under visible light irradiation. This work establishes surface-engineered SiNCs as a versatile platform for designing high-performance plasmonic–semiconductor photocatalysts.

## Introduction

Plasmonic metal nanoparticles (PMNPs) have emerged as promising photocatalysts, primarily due to their exceptional light-harvesting capabilities enabled via localized surface plasmon resonance (LSPR) (Jang et al., 2016; Linic et al., 2011; Linic et al., 2015). Interest in these materials extends beyond their optical properties to include unique physicochemical features such as morphology-dependent activity, operational versatility, and favorable cost-to-toxicity profiles (Adleman et al., 2009; da Silva et al., 2022; Herring and Montemore, 2023). These features have driven intense research into the development of PMNP-based heterogeneous photocatalysts (Gellé et al., 2020; Zhan et al., 2018; Zhan et al., 2020).

However, a critical limitation of freestanding PMNP-based photocatalyst is the rapid relaxation of photoexcited hot charge carriers through electron-electron scattering, which severely limits their direct catalytic activity (Brongersma et al., 2015; Ro et al., 2023). Hybridizing PMNPs with semiconducting materials offers a compelling strategy to circumvent this loss, enabling the transfer of plasmon-generated electrons across a metal-semiconductor interface (Clavero, 2014). In an ideal heterostructure, this interfacial charge injection, followed by migration over the Schottky barrier, can effectively suppress charge recombination and thus enhance photocatalytic efficiency (Moskovits, 2015). However, the practical performance of such hybrid structures is often hampered by two competing factors: the intrinsic rapid decay of plasmons and more importantly, weak electronic coupling at the heterointerface. This poor coupling can trap photoexcited carriers, confining them either to the bulk semiconductor or the PMNP surfaces, and diminishing the synergistic gains (Keller and Frontiera, 2017; Zhang Y. et al., 2018). To address these challenges, advanced interface engineering approaches aim to create ligand-free or atomically close contacts, which enable ultrafast charge transfer rates that can surpass carrier relaxation processes. Specifically, *in-situ* growth methods, where the semiconductor surface acts as a template for metal nucleation, have been shown to minimize interfacial barriers and maximize the efficiency of hot-electron injection (Celebi et al., 2021; Zhao et al., 2016).

In the past decade, significant effort has been devoted to developing synthetic routes for PMNP-semiconductor composites (Christopher and Moskovits, 2017; Wang et al., 2021; Zheng et al., 2015). However, the carrier-accepting capability of many semiconductor substrates is limited, which impedes effective charge separation and catalytic performance (Collado et al., 2018). Furthermore, synthetic protocols frequently rely on stabilizing agents to control PMNP growth, which leave residual organic ligands that mask catalytically active sites (Park et al., 2007; Quintanilla et al., 2010; Shan and Tenhu, 2007; Zhong et al., 2014). While post-synthetic treatments to remove these ligands exist, they are often complex and can compromise the integrity of the nanostructures (Aliaga et al., 2009; Cargnello et al., 2015; Lopez-Sanchez et al., 2011). Therefore, a straightforward approach to directly synthesize clean, unpassivated PMNPs on a compatible semiconductor substrate remains a desirable goal.

**SCHEME 1:**
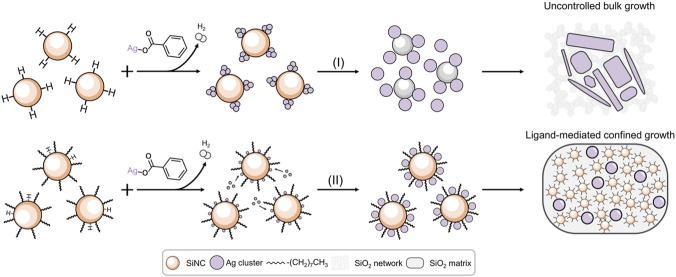
Schematic illustration of the synthesis of Ag/Si hybrid composites. Steps I depict the uncontrolled reduction leading to bulk Ag formation and SiNC oxidation. Steps II illustrate the confined growth of AgNPs mediated by surface-functionalized SiNCs, yielding a stable efficient hybrid dispersion.

To address this, we turn to silicon nanocrystals (SiNCs) as a versatile semiconductor platform. SiNCs offer not only size-tunable photophysical properties and relatively slower charge recombination due to their intrinsic indirect bandgap, but also highly tunable surface chemistry that can mediate redox reactions (Dasog et al., 2014; Sun et al., 2016; Veinot, 2006). This represents an opportunity to use the SiNC surface as a reductive platform for the *in-situ* growth of plasmonic metal nanoparticles (NPs). Specifically, we demonstrate a solution-processed, *in-situ* reduction method for fabricating colloidally stable silver nanoparticle/SiNC (AgNP/SiNC) hybrids. The surfaces of colloidally stable, surface-functionalized SiNCs were used as the platform, on which the Si-H bonds can effectively convert Ag ions to Ag atoms, and the alkyl ligands confine and regulate the growth of Ag nanoparticles (AgNPs). This enables precise control over the hybrids morphology and, ultimately, its photocatalytic function, as illustrated in Scheme 1. This engineered Ag@SiNC hybrid, featuring a clean and strongly coupled interface—defined as an intimate, ligand-free contact between AgNPs and SiNCs, free of insulating organic stabilizers or a thick native oxide layer—achieves enhanced catalytic performance for visible-light-driven CO_2_ reduction, delivering a CO yield of 1,552 μmol/g with near 100% selectivity.

## Materials and methods

### Chemicals

All chemicals used were commercially available and used without further purification: trimethoxysilane (TriMOS, 95%), hydrofluoric acid (HF, 49% aqueous solution, electronic grade), 2,2'-Azobis (2-methylpropionitrile) (AIBN, 98%), octene (99%), ethanol (95%), toluene (99.9%), triethanolamine (TEOA, 99%), acetonitrile (MeCN, 99%), nitric acid (68%), methanol (99.5%), and silver benzoate (99.99%) were purchased from Aladdin Chemical Inc.

### Preparation of oxide-embedded SiNCs

In a nitrogen-filled glovebox, 63 mmol of TriMOS (7.7 mg) was weighed and immediately transferred into a 100-mL Schlenk flask equipped with a magnetic stirrer. The flask was cooled in an ice bath to ∼0 °C. 10 mL of 68% nitric acid (90 mmol) and 10 mL of methanol (25 mmol) were added to the flask and mixed under continuous nitrogen flow. A clear, white gel formed within 5 min. The mixture was allowed to warm to room temperature and aged under a nitrogen atmosphere for 24 h. The product was isolated by vacuum filtration and dried in a vacuum oven for 16 h, yielding a blue powder. ∼1 g of this powder was placed in a quartz reaction boat and transferred to a high-temperature tube furnace (Lindberg). The sample was heated from ambient temperature to a peak temperature of 1,100 °C at a rate of 18 °C/min under a slightly reducing atmosphere (5% H_2_ + 95% Ar), held at 1,100 °C for 1 h, and cooled naturally to room temperature. The resulting amber-colored powder was manually ground using a mortar and pestle, then stored in a 20-mL vial under ambient conditions for further use.

### Liberation of hydride-terminated SiNCs (H-SiNCs)

A conventional HF-etching protocol was employed to release SiNCs from the SiO_2_ matrix (Clark et al., 2017). ∼0.5 g of the ground oxide-embedded SiNC powder was transferred to a polyethylene terephthalate (PTFE) beaker with a Teflon-coated stir bar. 5 mL of ethanol and 5 mL of deionized water were added to the beaker under mechanical stirring to form a brown suspension. Subsequently, 5 mL of HF aqueous solution (48%–51%) was added under stirring at room temperature to initiate the etching reaction (Caution! HF solution must be handled with extreme care). After 1 h, the suspension gradually turned orange. The H-SiNCs were extracted from the aqueous solution by multiple additions of toluene and then transferred to test tubes. The particles were isolated from the solution by centrifugation at 7,000 rpm for 10 min, re-dispersed in MeCN, and stored in vials for further use.

### Radical-initiated hydrosilylation for the preparation of alkyl-passivated SiNCs (alkyl-SiNCs)

Under a constant nitrogen flow, 1 mg of the radical initiator AIBN (4 μmol) and 20 mL of octene were added to the flask with mechanical stirring. The flask was heated to 65 °C in an oil bath, and the hydrosilylation reaction proceeded for 16 h, yielding a transparent orange solution. The product was transferred into 1.5 mL centrifuge tubes (∼0.3 mL per tube) and precipitated by adding a 1:1 (v/v) methanol/ethanol mixture (1.2 mL per tube). The precipitate was isolated by high-speed centrifugation at 17,000 rpm for 10 min. The supernatant was decanted, and the pellet was redispersed in a minimal amount of toluene. The precipitation/centrifugation cycle was repeated twice. The final alkyl-SiNC product was redispersed in MeCN and stored in vials for further use.

### Preparation of Ag@H-SiNCs

To the above-mentioned H-SiNCs MeCN dispersion (10 mg of H-SiNCs in 10 mL of MeCN), 200 μL of silver benzoate solution (2.3 wt% in MeCN) was added dropwise with stirring at room temperature. The reaction proceeded for 12 h, forming an opaque black suspension. The product was isolated by centrifugation (7,000 rpm, 10 min), washed by redispersion in MeCN (20 mL) and recentrifugation twice, dried under vacuum, and stored in a glass vial.

### Preparation of Ag@alkyl-SiNCs

To a dispersion of alkyl-SiNCs (10 mg in MeCN), 200 μL of a silver benzoate solution (2.3 wt% in MeCN) was added dropwise with stirring at room temperature. The reaction proceeded for 12 h, yielding a transparent orange colloidal dispersion consistent with the abovementioned alkyl-SiNCs. The product was transferred to 1.5-mL centrifuge tubes (∼0.3 mL per tube) and precipitated by adding a 1:1 (v/v) methanol/ethanol mixture (1.2 mL per tube), yielding a cloudy yellow suspension. The precipitate was isolated by centrifugation at 17,000 rpm for 10 min. The supernatant was decanted, and the particles were redispersed in a minimal amount of toluene. The suspension was centrifuged at 17,000 rpm for 10 min, and the pellet was redispersed in toluene and reprecipitated. This washing cycle was repeated twice. The final Ag@alkyl-SiNCs were redispersed in MeCN and stored in vials for further use.

### Photocatalytic CO_2_ reduction performance

Photocatalytic CO_2_ reduction was performed in a 50-mL sealed glass vial with magnetic stirring. 2.0 mg of powdery sample was suspended in 5 mL of a TEOA/MeCN mixed solvent of (1:4 v/v). The vial was pumped with CO_2_ (99.999%), sealed, and placed 5 cm above the light source (a 10 W light-emitting diode (LED), 10 W, λ = 450 nm, PCX-50B/50C, Beijing Perfectlight Technology Co., Ltd.). Reactions were conducted at room temperature for predetermined durations. The amount of CO produced was quantified using an off-line gas chromatograph (FuLi Analytical Instrument Co., Ltd., GC9790 plus) equipped with a thermal conductivity detector and nitrogen carrier gas.

### Carbon isotope tracer measurements

Isotope-labelling experiments were performed using ^13^CO_2_ under conditions identical to those described above. Briefly, 2 mg of Ag@alkyl-SiNCs sample was suspended in 5 mL of TEOA/MeCN (1:4 v/v) in a 50-mL glass vial. The headspace of the vial was purged three times (10 min each) with ^13^CO_2_ (99.999%, Sigma-Aldrich Inc.) to ensure a saturated ^13^CO_2_ atmosphere. The gaseous products were analyzed by gas chromatography-mass spectrometry (GCMS-QP2010, Shimadzu Inc.).

### Powdery X-ray diffraction (XRD) measurements

XRD patterns were acquired on a Rigaku SmartLab diffractometer (Bragg-Brentano geometry, Cu Kα1 radiation, *λ* = 1.54056 Å). Scans were performed from 2θ = 10°–80° with a total integration time of 350 min.

### X-ray photoelectron spectroscopic (XPS) measurements and analyses

XPS measurements were conducted using a Thermo Scientific K-Alpha system with a monochromatic Al Kα X-ray source (1,486.7 eV, spot size: 400 μm). Survey and high-resolution spectra were collected with a pass energy of 100 eV. Data were processed using CasaXPS software (VAMAS), with all spectra calibrated to the C *1s* emission at 284.8 eV.

### Absorption measurements

The absorption spectra were recorded on a Shimadzu spectrometer (UV-3600) using samples diluted in MeCN.

### Electron microscopy

High-angle annular dark-field scanning transmission electron microscopy (HAADF-STEM) images and energy-dispersive X-ray spectroscopy (EDX) results were acquired using an aberration-corrected Thermo Fisher Scientific Themis 60–300 microscope operated at an acceleration voltage of 200 kV with a probe current of ∼50 pA under high vacuum.

### Fourier-transform infrared (FT-IR) spectroscopy

FT-IR spectra were recorded on a Nicolet Nexus-670 spectrometer equipped with an ATR module, covering the range of 4,000–400 cm^-1^.

### Photoluminescence (PL) measurements

Steady-state PL spectra were recorded at room temperature using an FLS 980 spectrometer (Edinburgh Instruments) with a xenon lamp source (λ_ex_ = 365 nm). For time-resolved PL decays, ∼2 mL of SiNCs in chloroform solution was transferred in a quartz cuvette and excited by a 405 nm pulsed laser (2 W/cm^2^). The spectral resolution was ∼6 nm. Laser pulse width and repetition rate were set between 100–500 μs and 0.5–10 Hz, respectively. Emitted photons were collected with a 10 ×0.25 NA objective and directed to a 20 × 20 μm avalanche photodiode (Becker & Hickl, DPC-230) via a spectrometer (Andor Shamrock 500).

## Results and discussions

**FIGURE 1:**
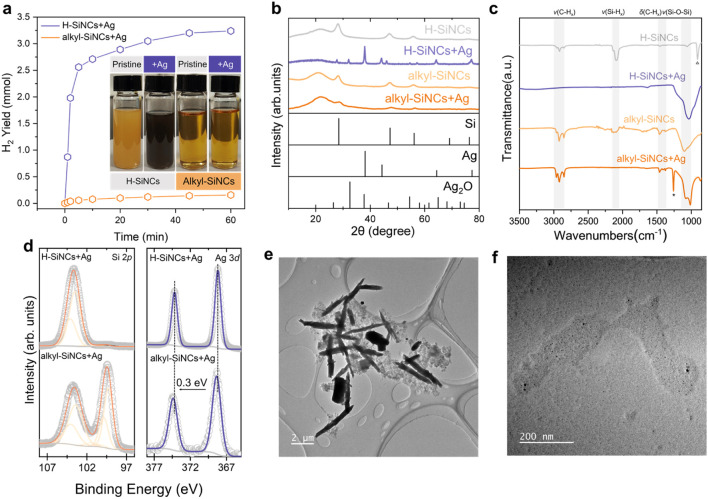
Materials characterization of Ag@SiNCs. **(a)** Hydrogen evolution profiles during the reaction of H-SiNCs and Alkyl-SiNCs with silver benzoate. Inset: photographs of colloidal dispersions of prestine SiNCs and the resulting Ag@SiNCs (solvent: MeCN). **(b)** Powder XRD patterns (Si: JCPDS No. 27‐1,402; Ag: JCPDS No.65-2871; Ag_2_O: JCPDS No.76–1,393), **(c)** FT-IR spectra, and **(d)** High-resolution X-ray photoelectron spectroscopic (XPS) results for pristine SiNCs and Ag@SiNCs. **(e)** Transmission electron microscopy (TEM) images of Ag@H-SiNCs and **(f)** Representative TEM image of Ag@alkyl-SiNCs, showing discrete AgNPs (5–20 nm) intimately associated with SiNCs.

We first prepared SiNCs with two distinct surface termination groups (hydride (Si-H) and alkyl groups) to serve as platforms for the subsequent AgNP growth (Islam et al., 2018). Upon reaction with silver benzoate, the H-SiNCs underwent a rapid redox process, evidenced by vigorous hydrogen evolution (Figure 1a). This uncontrolled reaction led to the formation of bulky silver structures and the complete oxidation of the SiNCs to SiO_2_ (Figure 1a). We hypothesized that tailoring the SiNC surface chemistry could provide precise control over the reaction pathway, enabling the synthesis of well-defined AgNPs/SiNC hybrids. To confirm this, we functionalized H-SiNCs via hydrosilylation with 1-octene via a radical initiation process to form alkyl-SiNCs (Yang et al., 2015). This surface modification drastically improved colloidal stability, yielding a transparent dispersion (inset, Figure 1a). The addition of a controlled amount of silver benzoate to these purified alkyl-SiNCs induced a mild and selective reduction of Ag^+^ ions, resulting in a homogeneous colloid where uniformly dispersed AgNPs coexisted on the surfaces of SiNCs. Notably, this process produced minimal hydrogen gas (Figure 1a). In addition, the colloidal stability of the alkyl-SiNC dispersion was preserved with no observable degradation under identical conditions (Figure 1a).

We next evaluated the structural integrity and the composition of the hybrid materials. Powder X-ray diffraction (XRD) was used to monitor crystallinity and phase composition (Figure 1b). Pristine SiNCs showed characteristic diffraction signals at 28.2^o^, 47.1^o^ and 55.8^o^, corresponding to the (111), (220), and (311) lattice planes of crystalline silicon (c-Si), respectively. After reaction with silver benzoate, these c-Si signal disappeared completely for the H-SiNCs sample, confirming its total oxidation. The XRD pattern for Ag@H-SiNCs was instead dominated by sharp signals from bulk Ag, with minor contributions from Ag_2_O due to oxidation. In contrast, the alkyl-SiNCs retained their distinct c-Si XRD signature after reaction. This confirms the successful preservation of the SiNC core alongside the controlled growth of AgNPs, resulting in a well-defined hybrid nanostructure. A slight of the c-Si signal intensity suggests partial surface oxidation, but no evidence of bulk Ag or silver oxide phases was detected.

FT-IR spectroscopy provided insight into surface chemistry (Figure 1c). For pristine H-SiNCs, the characteristic Si-H_*x*_ stretching absorption (∼2100 cm^-1^) was fully diminished during the interaction with Ag^+^, accompanied by the emergence of a strong Si-O-Si stretching band (∼1,050 cm^-1^), confirming oxidation. For alkyl-SiNCs and the resulting Ag@alkyl-SiNCs, the intense C-H_x_ stretching vibrations (2800–3,050 cm^-1^) remained unchanged, indicating the stability of the surface ligands. A weak residual Si-H_x_ signal present in the alkyl-SiNCs was also eliminated after reaction, indicating a similar reduction pathway that yields ligand-free AgNPs. No benzonate-related signals were detected, suggesting the absence of residual organic anions on AgNPs/SiNC hybrids after purification.

XPS provided additional information into elemental states and local chemical environments (Figure 1d). High-resolution Si 2*p* spectra showed the complete loss of the elemental Si(0) signal at ∼99.2 eV for Ag@H-SiNCs, consistent with the oxidation to SiO_x_ (0 < x ≤ 2). In contrast, this signal remained clearly visible in Ag@Alkyl-SiNCs, indicating the preservation of the c-Si core. High-resolution Ag 3*d* spectra displayed doublet peaks at 368.2 eV (Ag 3*d*_5/2_) and 374.3 eV (Ag 3*d*_3/2_) for both samples, verifying the presence of metallic Ag (i.e., Ag (0)). A slight positive shift (∼0.3 eV) and broadening of these Ag (0) peaks in the Ag@alkyl-SiNCs sample suggest a lower electron density on the AgNPs compared to bulk metal, indicative of electronic interaction at the Ag/Si interface (Liu et al., 2023). While atomic-scale imaging of the interface remains a challenge, the combined evidence from FT-IR (absence of organic residues) and XPS (electronic perturbation) strongly supports our assertion of a clean, electronically coupled Ag-Si interface. The retention of the crystalline Si XRD signature and Si(0) XPS signal in the Ag@alkyl-SiNCs after reaction confirms the effectiveness of the alkyl passivation layer in protecting the SiNC core from oxidation, a known vulnerability of unpassivated SiNCs.

TEM imaging revealed the distinct morphologies resulting from the two synthesis pathways. The Ag@H-SiNCs sample consisted of microscale flakes and rods embedded within a low-contrast matrix (Figure 1e), consistent with the formation of bulk silver domains and the oxidation of SiNC reflected by XRD results. In contrast, the Ag@alkyl-SiNC hybrid comparised discrete, high-contrast NPs (5–20 nm in diameter) embedded in a thinner oxide layer, where individual AgNPs and SiNCs can be differentiated based on their relative contrast (Figure 1f).

**FIGURE 2:**
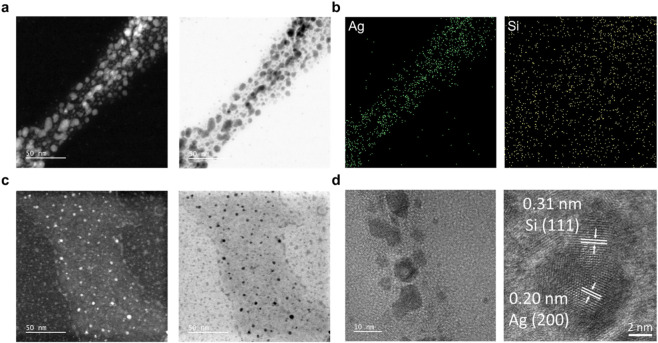
Electron microscopy analysis of Ag@alkyl-SiNCs. **(a)** Bright-field and dark-field TEM images of Ag@alkyl-SiNCs and **(b)** Corresponding HAADF-STEM image and EDS elemental maps showing the distribution of Si (yellow) and Ag (green), confirming the intimate hybrid structure; **(c)** Bright-field and dark-field TEM images of Ag@alkyl-SiNC from a separate region. **(d)** HRTEM image of the Ag@alkyl-SiNC hybrid structure, showing distinct lattice fringes from (200) planes of the face-centered cubic (fcc) Ag and c-Si.

Combined bright-field and dark-field TEM imaging, coupled with high-resolution analysis, elucidated the detailed structure and elemental distribution of the Ag@alkyl-SiNC hybrid (Figures 2a,b). The images confirm the close connection between AgNPs and SiNCs. Energy-dispersive X-ray spectroscopy (EDS) mapping showed uniform Si distribution, indicating a widespread dispersion of SiNCs/SiO_x_, while the Ag signals were localized in discrete regions colocalized with Si, confirming the coexistence of Ag and Si domains (Figure 2b). High-resolution TEM (HRTEM) images showed distinct lattice fringes with spacings of 0.20 nm and 0.31 nm (Figures 2c,d), corresponding to the (200) planes of the face-centered cubic (fcc) Ag and the (111) planes of c-Si, respectively (Panthani et al., 2012; Vidyasagar et al., 2023).

**FIGURE 3:**
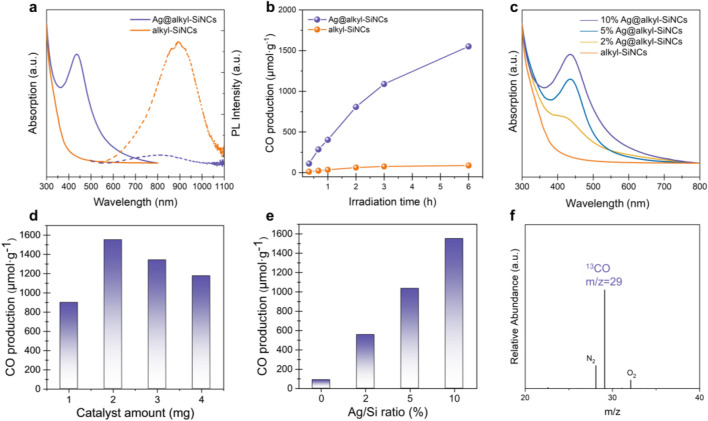
Photocatalytic CO_2_ reduction performance of Ag@alkyl-SiNCs. **(a)** Absorption and PL spectra of pristine alkyl-SiNCs and Ag@alkyl-SiNCs hybrid. **(b)** CO production yield as a function of irradiation time in a CO_2_-saturated solution under a 450 nm blue LED. **(c)** Absorption spectra of pristine SiNCs and Ag@alkyl-SiNCs with various Ag/Si ratios. **(d)** Effect of catalyst loading and **(e)** Ag/Si amtomic ratio on the CO production rate. **(f)** Gas chromatography-mass spectrometry (GC-MS) analysis of the gas phase after photocatalytic reduction of ^13^CO_2_, confirming the production of ^13^CO. Signals for O_2_ and N_2_ are attributed to minor atmospheric leakage during injection.

We further investigated the photocatalytic performance of the AgNP/SiNC hybrids for CO_2_ reduction under visible-light irradiation. As for the standard reaction, 2 mg of powdery Ag@Alkyl-SiNC power was dispersed in a CO_2_-saturated MeCN/TEOA solution (see Materials and methods section above for more details). Upon irradiation with a 450 nm blue LED, CO was formed immediately, with no activity detected in the dark. The enhanced catalytic activity is attributed to the synergistic interaction between the components.: the intense light absorption by the Ag-incorporated SiNCs promotes and enhances the LSPR of the adjacent AgNPs. The absorption spectra in Figure 3a confirmed this, showing that the Ag@alkyl-SiNC hybrid exhibits a distinct LSPR peak at 450 nm, which is absent in the pristine alkyl-SiNCs (Campos et al., 2019). In the meantime, the significant quenching of the SiNC PL in the hybrid (Figure 3a) points to efficient suppression of radiative recombination, which is consistent with rapid extraction of photo-generated electrons from SiNCs by the AgNPs.

Under optimized conditions (5 mL of MeCN, 1 mL of TEOA, 2 mg of Ag@Alkyl-SiNCs), a CO production yield of 1,552 μmol/g was achieved after 6 h of continuous irradiation (Figure 3b). As summarized in Table 1, the CO production rate of our Ag@alkyl-SiNCs compares favorably with many recently reported metal/semiconductor hybrid photocatalysts, including those based on g-C_3_N_4_ and perovskites.

**TABLE 1:** Comparison of photocatalytic CO_2_ reduction performance of Ag@alkyl-SiNCs with representative recently reported photocatalysts.

Photocatalyst system	Normalized CO yield (μmol·g^-1^h^-1^)	Product	Light source	Ref.
Ag@alkyl-SiNCs	258.6	CO	450 LED	This work
Cs_2_AgBiBr_6_-1GCN	12.14	CO, CH_4_	250 W Hg Lamp	Purohit et al. (2023)
g-C_3_N_4_/Bi_2_WO_6_	17.78	CO, CH_4_	Visible Light	Chen et al., (2025)
TiO_2_/CsPbBr_3_ nanofibers	9.02	CO	Visible Light	Xu et al. (2020)
CsPbBr_3_-Ni(tpy)	287.3	CO, CH_4_	Visible Light	Chen et al. (2020)

We attribute the enhanced photocatalytic activity to a hot-electron transfer mechanism, a process extensively studied in pioneering works on plasmonic photocatalysis (Amirja et al., 2023; Zhang X. L. et al., 2018). In this model, the LSPR excitation of AgNPs generates a non-thermal distribution of hot electrons, which can be injected into the adjacent SiNCs before thermalizing via electron-electron scattering. While direct observation of this ultrafast process requires techniques such as transient absorption spectroscopy, our steady-state observations—namely, the appearance of the LSPR peak, the quenching of SiNC photoluminescence, and the shift in Ag 3*d* XPS binding energy—are all consistent with this proposed charge transfer pathway and the resulting suppression of recombination.

**FIGURE 4:**
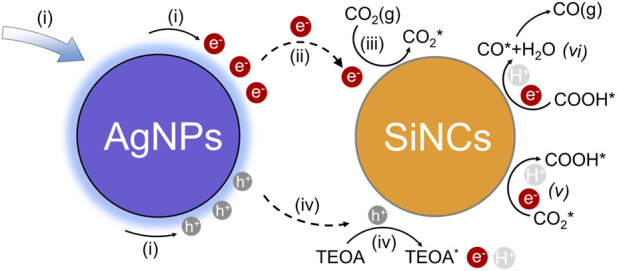
Proposed mechanism for plasmon-enhanced photocatalytic CO_2_ reduction over the Ag@Si nanohybrid. (i) LSPR excitation and hot carrier generation; (ii) hot electron injection and charge separation; (iii) CO_2_ adsorption and activation; (iv) sacrificial agent scavenging; (v) key COOH intermediate formation; (vi) CO formation and CO desorption. (e^−^, h^+^ denote photogenerated electrons and holes, respectively. H^+^ represent protons supplied from the oxidative decomposition of TEOA. The asterisk (*) indicates an active site or an adsorbed species on the catalyst surface).

We further optimized catalyst loading and the Ag/Si ratio, identifying 2 mg of hybrid materials with a Ag content of 10 at% Ag as the optimal formulation in this content range (Figures 3c–e). We ascribe this optimum to a trade-off between light harvesting and carrier utilization: at this ratio, the density of plasmonic hot spots is sufficient to maximize hot-electron generation and interfacial injection, while avoiding the parasitic light shielding and charge recombination centers typically associated with excessive metal coverage (Hou and Cronin, 2013; Li et al., 2022; Zhang et al., 2013). Isotopic labeling experiments using ^13^CO_2_ confirmed that the generated CO originated exclusively from the supplied CO_2_ feedstock (Figure 3f). These characterizations and performance evaluations provide a comprehensive understanding of the CO_2_ reduction mechanism summarized in Figure 4. The process initiates with the photoexcitation of LSPR in Ag nanoparticles under visible-light irradiation (i), generating energetic hot electron-hole pairs. Subsequently, the hot electrons are efficiently injected into the SiNCs (ii), a critical step that spatially separates the photogenerated charge carriers and mitigates recombination. The hot electrons on SiNCs then facilitate the adsorption and activation of CO_2_ molecules on their surfaces (iii), activating the linear CO_2_ structure and lowering the energy barrier for reduction. Following activation, the holes left on the AgNPs and oxidized the sacrificial agent triethanolamine (TEOA) (iv). This oxidative decomposition of TEOA provides both the protons required for CO_2_ reduction and electrons to balance the charge, ultimately being consumed to drive the photocatalytic cycle forward. A proton-coupled electron transfer process occurs, wherein the activated CO_2_ interacts with protons supplied by the sacrificial agent TEOA to form the key carboxyl intermediate (*COOH) (v). This intermediate undergoes further reduction and dehydration, leading to (vi) the *CO formation and eventual desorption of CO as gaseous CO product (Chen et al., 2023; Jin et al., 2023; Liao et al., 2024; Liu et al., 2017; Zhou and Xia, 2024). This mechanism highlights the important roles of surface-anchored AgNPs as plasmonic antenna for visible light harvesting and hot-electron generation, and of surface-tailored SiNCs as robust platforms for electron transfer, CO_2_ activation, and surface catalysis, with their intimate interface being essential for the observed high activity and selectivity.

## Conclusion

In summary, we have developed a facile solution-phase strategy for *in-situ* synthesis of ligand-free AgNPs anchored to functionalized SiNCs. We demonstrate that SiNC surface chemistry is key to regulating AgNP growth while preventing oxidation of the c-Si core. When applied to photocatalytic CO_2_ reduction, the Ag@alkyl-SiNC hybrid exhibits a pronounced synergistic effect, where SiNC-enhanced light absorption promotes plasmonic response of AgNPs, facilitating efficient charge separation. This mechanism delivers a high CO yield of 1,552 μmol/g with near-unity selectivity under blue-light irradiation, highlighting interface engineering as a critical design principle for metal-semiconductor photocatalysts. Future studies employing techniques such as UPS and TRPL could quantitatively elucidate the band alignment, providing deeper insights into interfacial energetics. Moreover, upon validation of long-term stability, this material offers significant promise as a commercial catalyst for diverse redox reactions, owing to its ligand-free, clean interface that eliminates site-blocking stabilizers, and its cost-effectiveness derived from earth-abundant silicon and silver precursors—positioning it as a sustainable and scalable platform for practical catalytic applications.

## Data Availability

The original contributions presented in the study are included in the article/supplementary material, further inquiries can be directed to the corresponding authors.
